# Poly[4-(dimethyl­amino)pyridinium [(μ_6_-5-carboxy­benzene-1,2,4-tricarboxy­ato-κ^6^ *O* ^1^:*O* ^1′^:*O* ^2^:*O* ^4^:*O* ^4′^:*O* ^5^)diargentate(I)]]

**DOI:** 10.1107/S1600536809028839

**Published:** 2009-07-25

**Authors:** Xiao-Fei Zhu, Yan-Hong Zhou, Li Guan, Hong Zhang

**Affiliations:** a Faculty of Chemistry, Northeast Normal University, Changchun 130024, People’s Republic of China

## Abstract

In the title compound, {(C_7_H_11_N_2_)[Ag_2_(C_10_H_3_O_8_)]}_*n*_, the polymeric anion consists of two Ag^I^ atoms and a Hbtc^3−^ ligand (H_4_btc = benzene-1,2,4,5-tetra­carboxylic acid). Each Ag^I^ atom is coordinated by four O atoms from three different Hbtc^3−^ ligands. The two Ag^I^ atoms are bridged by two bidentate carboxyl­ate groups into an Ag_2_O_4_ cyclic unit, with an Ag⋯Ag distance of 2.8189 (3) Å. In this way, the Ag atoms are connected by the Hbtc^3−^ ligands into an extended two-dimensional layer structure. A three-dimensional network is accomplished through O—H⋯O hydrogen bonds between the anionic layers. The cationic guest Hdmap^+^ [dmap = 4-(dimethyl­amino)pyridine] is trapped in the network and adheres to the layer by an N—H⋯O hydrogen bond.

## Related literature

For general background to metal-organic frameworks with 1,2,4,5-benzene­tetra­carboxyl­ate liganda, see: Cao *et al.* (2002 ▶); Hu *et al.* (2004 ▶); Li *et al.* (2003 ▶). For related complexes, see: Chen (2008 ▶); Sun *et al.* (2003 ▶); Zheng *et al.* (2002 ▶, 2003 ▶).
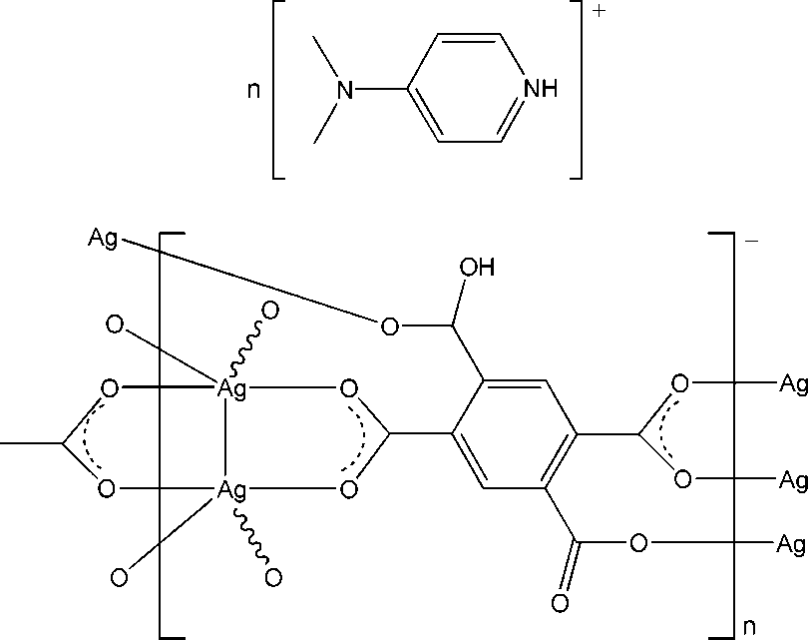


## Experimental

### 

#### Crystal data

(C_7_H_11_N_2_)[Ag_2_(C_10_H_3_O_8_)]
*M* *_r_* = 590.04
Triclinic, 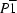

*a* = 9.7192 (3) Å
*b* = 9.9936 (5) Å
*c* = 10.4968 (3) Å
α = 113.304 (4)°
β = 97.140 (3)°
γ = 103.260 (3)°
*V* = 884.65 (7) Å^3^
*Z* = 2
Mo *K*α radiation
μ = 2.27 mm^−1^
*T* = 293 K
0.24 × 0.18 × 0.14 mm

#### Data collection

Oxford Diffraction Gemini R Ultra diffractometer
Absorption correction: multi-scan (*CrysAlis RED*; Oxford Diffraction, 2006 ▶) *T*_min_ = 0.611, *T*_max_ = 0.725
7226 measured reflections
3124 independent reflections
2808 reflections with *I* > 2σ*I*)
*R*_int_ = 0.012

#### Refinement

*R*[*F* ^2^ > 2σ(*F* ^2^)] = 0.018
*wR*(*F* ^2^) = 0.046
*S* = 1.06
3124 reflections
265 parameters
H-atom parameters constrained
Δρ_max_ = 0.35 e Å^−3^
Δρ_min_ = −0.47 e Å^−3^

### 

Data collection: *CrysAlis CCD* (Oxford Diffraction, 2006 ▶); cell refinement: *CrysAlis RED* (Oxford Diffraction, 2006 ▶); data reduction: *CrysAlis RED*; program(s) used to solve structure: *SHELXS97* (Sheldrick, 2008 ▶); program(s) used to refine structure: *SHELXL97* (Sheldrick, 2008 ▶); molecular graphics: *DIAMOND* (Brandenburg, 1999 ▶); software used to prepare material for publication: *SHELXL97*.

## Supplementary Material

Crystal structure: contains datablocks I, global. DOI: 10.1107/S1600536809028839/hy2204sup1.cif

Structure factors: contains datablocks I. DOI: 10.1107/S1600536809028839/hy2204Isup2.hkl

Additional supplementary materials:  crystallographic information; 3D view; checkCIF report

## Figures and Tables

**Table 1:** Selected bond lengths (Å)

Ag1—O1	2.5220 (15)
Ag1—O3^i^	2.1784 (15)
Ag1—O3	2.7573 (19)
Ag1—O6^ii^	2.1765 (15)
Ag2—O4	2.2091 (15)
Ag2—O5^iii^	2.2224 (16)
Ag2—O5^iv^	2.873 (2)
Ag2—O7^iv^	2.4442 (15)

Symmetry codes: (i) 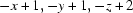; (ii) 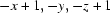; (iii) 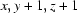; (iv) 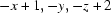.

**Table 2:** Hydrogen-bond geometry (Å, °)

*D*—H⋯*A*	*D*—H	H⋯*A*	*D*⋯*A*	*D*—H⋯*A*
N2—H⋯O7^v^	0.84	1.88	2.720 (2)	177
O2—H2⋯O8^vi^	0.82	1.73	2.541 (2)	173

Symmetry codes: (v) 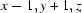; (vi) 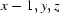.

**Fig. 1.**
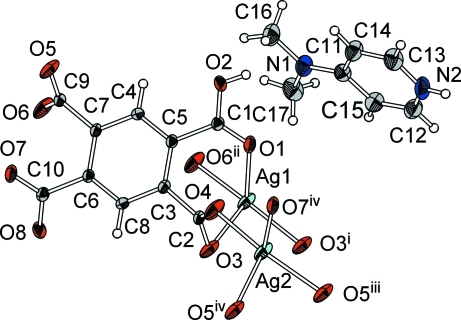
The asymmetric unit of the title compound. Displacement ellipsoids are drawn at the 50% probability level. [Symmetry codes: (i) 1 - x, 1 - y, 2 - z; (ii) 1 - x, -y, 1 - z; (iii) x, 1 + y, 1 + z; (iv) 1 - x, -y, 2 - z.]

**Fig. 2.**
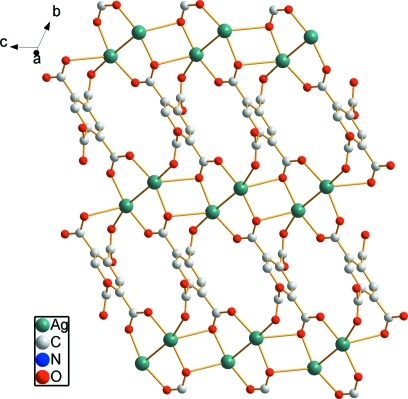
A view of the two-dimensional layer in the title compound. H atoms are omitted for clarity.

## supplementary crystallographic information

### Comment

More efforts have been made to construct MOFs (metal organic frameworks) materials by using 1,2,4,5-benzenetetracarboxylic acid (H_4_btc) as molecular building block, owing to its complexed coordination modes to metal ions and various dimensionalities (Cao *et al.*, 2002; Hu *et al.*, 2004; Li *et al.*, 2003). According to literature, the combination of H_4_btc, as a polydentate ligand and silver(I) can produce various architectures, involving in [Ag_2_(pbi)_2_(H_2_btc)]_n_ [pbi = 2-(3-pyridyl)-1*H*-benzimidazole] (Chen, 2008), [Ag(µ_3_-hmt)]_2_[Ag(NH_3_)_2_]_2_(btc).3H_2_O (hmt = hexamethylenetetramine) (Zheng *et al.*, 2002), [Ag_8_(µ_3_-hmt)_2_(µ_4_-hmt)_2_(µ-btc)_2_(µ-H_2_O)_3_].18H_2_O (Zheng *et al.*, 2003), and [Ag(bipy)][H_2_btc]_0.5_.H_2_O (Sun *et al.*, 2003). Herein, the title complex, [Hdmap][Ag_2_(Hbtc)] (dmap = 4-dimethylaminopyridine), with a layer structure is reported.

The structure of the title compound contains two crystallographically independent Ag^I^ atoms, one (Hbtc)^3-^ ligand and one (Hdmap)^+^ cantion. Each Ag^I^ atom is coordinated by four carboxylate O atoms from three different Hbtc ligands (Fig. 1), with three close bond distances [average Ag1—O = 2.2923 (15) and Ag2—O = 2.2919 (15) Å] and one long bond distance [Ag1—O = 2.7573 (19) and Ag2—O = 2.873 (2) Å] (Table 1). It is worth noting that two adjacent Ag1 and Ag2 atoms are bridged by two bidentate carboxylate groups into an Ag_2_O_4_ cyclic unit, with an Ag···Ag distance of 2.8189 (3) Å. The Hbtc ligand connects six Ag atoms, leading to a two-dimensional anionic layer (Fig. 2). The interlayer O—H···O hydrogen bonds hold adjacent layers together to bring out a supramolecular network. The cationic guest (Hdmap)^+^ is trapped in the network and adhere to the layer by an N—H···O hydrogen bond (Table 2).

### Experimental

A mixture of pyromelitic acid anhydride (0.218 g, 0.1 mmol) in distilled water (10 ml) was stirred at 333 K for 1 h until to get clear solution and then a DMF solution (2 ml) of AgNO_3_ (0.169 g, 0.1 mmol) was added on stirring for 1 h under ambient condition. The resulting solution was allowed to stand in air at room temperature for 3 d. Colorless crystals were collected in 77.8% yield based on AgNO_3_.

### Refinement

H atoms were positioned geometrically and refined as riding atoms, with C—H = 0.93 (aromatic) and 0.96 (methyl), N—H = 0.84 and O—H = 0.82 Å, and with *U*_iso_(H) = 1.2(or 1.5 for methyl and hydroxyl)*U*_eq_(C, N, O).

### Crystal data

**Table:**

(C_7_H_11_N_2_)[Ag_2_(C_10_H_3_O_8_)]	*Z* = 2
*M**_r_* = 590.04	*F*(000) = 576
Triclinic, *P*1	*D*_x_ = 2.215 Mg m^−^^3^
Hall symbol: -P 1	Mo *K*α radiation, λ = 0.71073 Å
*a* = 9.7192 (3) Å	Cell parameters from 3901 reflections
*b* = 9.9936 (5) Å	θ = 4.4–25.0°
*c* = 10.4968 (3) Å	µ = 2.27 mm^−^^1^
α = 113.304 (4)°	*T* = 293 K
β = 97.140 (3)°	Block, colorless
γ = 103.260 (3)°	0.24 × 0.18 × 0.14 mm
*V* = 884.65 (7) Å^3^	

### Data collection

**Table:**

Oxford Diffraction Gemini R Ultra diffractometer	3124 independent reflections
Radiation source: fine-focus sealed tube	2808 reflections with *I* > 2σ(*I*)
graphite	*R*_int_ = 0.012
Detector resolution: 10.0 pixels mm^-1^	θ_max_ = 25.0°, θ_min_ = 4.4°
ω scans	*h* = −11→11
Absorption correction: multi-scan (*CrysAlis RED*; Oxford Diffraction, 2006)	*k* = −11→11
*T*_min_ = 0.611, *T*_max_ = 0.725	*l* = −12→12
7226 measured reflections	

### Refinement

**Table:**

Refinement on *F*^2^	Secondary atom site location: difference Fourier map
Least-squares matrix: full	Hydrogen site location: inferred from neighbouring sites
*R*[*F*^2^ > 2σ(*F*^2^)] = 0.018	H-atom parameters constrained
*wR*(*F*^2^) = 0.046	*w* = 1/[σ^2^(*F*_o_^2^) + (0.0251*P*)^2^ + 0.4969*P*] where *P* = (*F*_o_^2^ + 2*F*_c_^2^)/3
*S* = 1.06	(Δ/σ)_max_ = 0.003
3124 reflections	Δρ_max_ = 0.35 e Å^−^^3^
265 parameters	Δρ_min_ = −0.47 e Å^−^^3^
0 restraints	Extinction correction: *SHELXL97* (Sheldrick, 2008), Fc^*^=kFc[1+0.001xFc^2^λ^3^/sin(2θ)]^-1/4^
Primary atom site location: structure-invariant direct methods	Extinction coefficient: 0.0157 (6)

### Fractional atomic coordinates and isotropic or equivalent isotropic displacement parameters (Å^2^)

**Table:**

	*x*	*y*	*z*	*U*_iso_*/*U*_eq_	
Ag1	0.421397 (19)	0.410977 (18)	0.802682 (17)	0.02690 (8)	
Ag2	0.421385 (19)	0.385653 (19)	1.290598 (17)	0.02889 (9)	
O1	0.22857 (16)	0.20334 (17)	0.81649 (18)	0.0277 (4)	
O2	0.10698 (15)	−0.04467 (17)	0.71974 (19)	0.0329 (4)	
H2	0.0364	−0.0126	0.7220	0.049*	
O3	0.57621 (18)	0.37804 (17)	1.02125 (17)	0.0326 (4)	
O4	0.4252 (2)	0.21751 (18)	1.07819 (17)	0.0344 (4)	
O5	0.41174 (19)	−0.39642 (18)	0.45762 (18)	0.0357 (4)	
O6	0.5636 (2)	−0.23946 (18)	0.39821 (17)	0.0341 (4)	
O7	0.75751 (16)	−0.21312 (17)	0.65497 (17)	0.0251 (3)	
O8	0.87465 (16)	0.03113 (17)	0.7214 (2)	0.0336 (4)	
C1	0.2265 (2)	0.0702 (2)	0.7685 (2)	0.0178 (4)	
C2	0.4977 (2)	0.2487 (2)	0.9985 (2)	0.0173 (4)	
C3	0.4930 (2)	0.1181 (2)	0.8600 (2)	0.0160 (4)	
C4	0.3628 (2)	−0.0996 (2)	0.6355 (2)	0.0177 (4)	
H4	0.2755	−0.1617	0.5684	0.021*	
C5	0.3627 (2)	0.0251 (2)	0.7570 (2)	0.0160 (4)	
C6	0.6223 (2)	−0.0377 (2)	0.7128 (2)	0.0157 (4)	
C7	0.4913 (2)	−0.1332 (2)	0.6121 (2)	0.0153 (4)	
C8	0.6218 (2)	0.0862 (2)	0.8348 (2)	0.0185 (4)	
H8	0.7093	0.1493	0.9012	0.022*	
C9	0.4888 (2)	−0.2673 (2)	0.4775 (2)	0.0168 (4)	
C10	0.7617 (2)	−0.0765 (2)	0.6939 (2)	0.0192 (4)	
N1	0.0720 (2)	0.4030 (2)	0.1392 (2)	0.0333 (5)	
N2	−0.1309 (2)	0.6318 (3)	0.4369 (2)	0.0378 (5)	
H	−0.1683	0.6777	0.5021	0.045*	
C11	0.0048 (2)	0.4765 (3)	0.2354 (2)	0.0271 (5)	
C12	−0.0362 (3)	0.7091 (3)	0.3890 (3)	0.0354 (6)	
H12	−0.0172	0.8144	0.4240	0.042*	
C13	−0.1624 (3)	0.4795 (3)	0.3866 (3)	0.0375 (6)	
H13	−0.2296	0.4279	0.4204	0.045*	
C14	−0.0985 (3)	0.3988 (3)	0.2872 (3)	0.0343 (6)	
H14	−0.1223	0.2931	0.2533	0.041*	
C15	0.0325 (3)	0.6369 (3)	0.2906 (3)	0.0333 (6)	
H15	0.0981	0.6930	0.2592	0.040*	
C16	0.0426 (3)	0.2378 (3)	0.0780 (3)	0.0407 (6)	
H16A	−0.0540	0.1883	0.0172	0.061*	
H16B	0.0505	0.2065	0.1535	0.061*	
H16C	0.1119	0.2095	0.0231	0.061*	
C17	0.1797 (3)	0.4867 (4)	0.0902 (3)	0.0423 (7)	
H17A	0.2588	0.5591	0.1700	0.063*	
H17B	0.1351	0.5400	0.0473	0.063*	
H17C	0.2160	0.4161	0.0210	0.063*	

### Atomic displacement parameters (Å^2^)

**Table:**

	*U*^11^	*U*^22^	*U*^33^	*U*^12^	*U*^13^	*U*^23^
Ag1	0.03846 (13)	0.01606 (11)	0.01917 (11)	0.00464 (7)	0.01347 (8)	0.00093 (7)
Ag2	0.04268 (13)	0.01682 (11)	0.02185 (11)	0.00567 (8)	0.01695 (8)	0.00245 (8)
O1	0.0209 (8)	0.0163 (8)	0.0436 (10)	0.0084 (6)	0.0142 (7)	0.0076 (7)
O2	0.0123 (7)	0.0184 (8)	0.0581 (11)	0.0053 (6)	0.0090 (7)	0.0065 (8)
O3	0.0409 (10)	0.0154 (8)	0.0288 (9)	0.0006 (7)	0.0191 (7)	−0.0016 (7)
O4	0.0535 (11)	0.0211 (8)	0.0240 (8)	0.0075 (7)	0.0231 (8)	0.0037 (7)
O5	0.0431 (10)	0.0148 (8)	0.0356 (9)	−0.0001 (7)	0.0255 (8)	−0.0019 (7)
O6	0.0553 (11)	0.0201 (8)	0.0233 (8)	0.0081 (7)	0.0245 (8)	0.0034 (7)
O7	0.0233 (8)	0.0173 (8)	0.0361 (9)	0.0103 (6)	0.0155 (7)	0.0084 (7)
O8	0.0143 (8)	0.0191 (8)	0.0614 (12)	0.0046 (6)	0.0133 (7)	0.0108 (8)
C1	0.0168 (10)	0.0168 (11)	0.0191 (10)	0.0056 (8)	0.0084 (8)	0.0058 (9)
C2	0.0181 (10)	0.0166 (10)	0.0149 (9)	0.0087 (8)	0.0047 (8)	0.0027 (8)
C3	0.0185 (10)	0.0132 (10)	0.0154 (10)	0.0056 (8)	0.0061 (8)	0.0045 (8)
C4	0.0146 (10)	0.0155 (10)	0.0173 (10)	0.0036 (8)	0.0026 (8)	0.0026 (8)
C5	0.0151 (10)	0.0132 (10)	0.0199 (10)	0.0049 (7)	0.0079 (8)	0.0061 (8)
C6	0.0144 (10)	0.0132 (10)	0.0185 (10)	0.0047 (7)	0.0067 (8)	0.0050 (8)
C7	0.0173 (10)	0.0130 (9)	0.0160 (9)	0.0063 (8)	0.0073 (8)	0.0049 (8)
C8	0.0155 (10)	0.0143 (10)	0.0187 (10)	0.0028 (8)	0.0031 (8)	0.0016 (8)
C9	0.0168 (10)	0.0164 (10)	0.0150 (10)	0.0071 (8)	0.0035 (8)	0.0036 (8)
C10	0.0181 (10)	0.0173 (11)	0.0199 (10)	0.0063 (8)	0.0074 (8)	0.0045 (9)
N1	0.0372 (11)	0.0354 (11)	0.0322 (11)	0.0154 (9)	0.0194 (9)	0.0141 (9)
N2	0.0413 (12)	0.0458 (13)	0.0318 (11)	0.0272 (10)	0.0174 (10)	0.0123 (10)
C11	0.0270 (12)	0.0322 (13)	0.0235 (11)	0.0108 (10)	0.0076 (9)	0.0122 (10)
C12	0.0420 (14)	0.0289 (13)	0.0338 (13)	0.0143 (11)	0.0061 (11)	0.0113 (11)
C13	0.0364 (14)	0.0422 (15)	0.0414 (14)	0.0136 (11)	0.0216 (12)	0.0213 (13)
C14	0.0383 (14)	0.0280 (13)	0.0401 (14)	0.0111 (11)	0.0188 (12)	0.0150 (12)
C15	0.0369 (13)	0.0308 (13)	0.0344 (13)	0.0097 (10)	0.0128 (11)	0.0156 (11)
C16	0.0437 (15)	0.0410 (16)	0.0369 (14)	0.0218 (12)	0.0152 (12)	0.0102 (13)
C17	0.0371 (14)	0.0576 (18)	0.0399 (15)	0.0176 (13)	0.0225 (12)	0.0236 (14)

### Geometric parameters (Å, °)

**Table:**

Ag1—O1	2.5220 (15)	C6—C8	1.387 (3)
Ag1—O3^i^	2.1784 (15)	C6—C7	1.400 (3)
Ag1—O3	2.7573 (19)	C6—C10	1.507 (3)
Ag1—O6^ii^	2.1765 (15)	C7—C9	1.507 (3)
Ag2—O4	2.2091 (15)	C8—H8	0.9300
Ag2—O5^iii^	2.2224 (16)	N1—C11	1.336 (3)
Ag2—O5^iv^	2.873 (2)	N1—C16	1.455 (3)
Ag2—O7^iv^	2.4442 (15)	N1—C17	1.460 (3)
Ag1—Ag2^i^	2.8189 (3)	N2—C12	1.339 (3)
O1—C1	1.216 (3)	N2—C13	1.341 (3)
O2—C1	1.306 (2)	N2—H	0.84
O2—H2	0.82	C11—C15	1.417 (3)
O3—C2	1.252 (3)	C11—C14	1.420 (3)
O4—C2	1.244 (3)	C12—C15	1.355 (3)
O5—C9	1.255 (3)	C12—H12	0.9300
O6—C9	1.240 (3)	C13—C14	1.361 (3)
O7—C10	1.249 (3)	C13—H13	0.9300
O8—C10	1.257 (2)	C14—H14	0.9300
C1—C5	1.496 (3)	C15—H15	0.9300
C2—C3	1.508 (3)	C16—H16A	0.9600
C3—C8	1.392 (3)	C16—H16B	0.9600
C3—C5	1.401 (3)	C16—H16C	0.9600
C4—C5	1.387 (3)	C17—H17A	0.9600
C4—C7	1.392 (3)	C17—H17B	0.9600
C4—H4	0.9300	C17—H17C	0.9600
			
O6^ii^—Ag1—O3^i^	165.22 (6)	C4—C7—C6	118.95 (18)
O6^ii^—Ag1—O1	88.08 (6)	C4—C7—C9	120.01 (17)
O3^i^—Ag1—O1	104.84 (6)	C6—C7—C9	120.99 (17)
O6^ii^—Ag1—Ag2^i^	82.64 (4)	C6—C8—C3	121.33 (18)
O3^i^—Ag1—Ag2^i^	83.04 (4)	C6—C8—H8	119.3
O1—Ag1—Ag2^i^	162.98 (4)	C3—C8—H8	119.3
O3—Ag1—O1	80.23 (5)	O6—C9—O5	126.56 (19)
O3—Ag1—O3^i^	82.06 (6)	O6—C9—C7	117.07 (18)
O3—Ag1—O6^ii^	107.63 (5)	O5—C9—C7	116.36 (17)
O3—Ag1—Ag2^i^	116.16 (4)	O7—C10—O8	124.57 (19)
O4—Ag2—O5^iii^	158.69 (7)	O7—C10—C6	117.63 (17)
O4—Ag2—O7^iv^	97.68 (6)	O8—C10—C6	117.78 (18)
O5^iii^—Ag2—O7^iv^	97.08 (5)	C11—N1—C16	122.5 (2)
O4—Ag2—Ag1^i^	81.08 (4)	C11—N1—C17	120.8 (2)
O5^iii^—Ag2—Ag1^i^	81.40 (4)	C16—N1—C17	116.8 (2)
O7^iv^—Ag2—Ag1^i^	168.58 (4)	C12—N2—C13	120.7 (2)
O5^iv^—Ag2—O4	119.23 (5)	C12—N2—H	120.9
O5^iv^—Ag2—O5^iii^	78.86 (6)	C13—N2—H	118.3
O5^iv^—Ag2—O7^iv^	78.31 (5)	N1—C11—C15	121.4 (2)
O5^iv^—Ag2—Ag1^i^	112.29 (4)	N1—C11—C14	122.1 (2)
C1—O1—Ag1	123.41 (13)	C15—C11—C14	116.4 (2)
C1—O2—H2	109.5	N2—C12—C15	121.3 (2)
C2—O3—Ag1^i^	123.07 (13)	N2—C12—H12	119.3
C2—O4—Ag2	124.47 (14)	C15—C12—H12	119.3
C9—O5—Ag2^v^	123.12 (13)	N2—C13—C14	121.5 (2)
C9—O6—Ag1^ii^	124.62 (14)	N2—C13—H13	119.3
C10—O7—Ag2^iv^	121.06 (12)	C14—C13—H13	119.3
O1—C1—O2	123.50 (18)	C13—C14—C11	119.7 (2)
O1—C1—C5	122.02 (17)	C13—C14—H14	120.1
O2—C1—C5	114.46 (17)	C11—C14—H14	120.1
O4—C2—O3	126.86 (19)	C12—C15—C11	120.3 (2)
O4—C2—C3	117.34 (18)	C12—C15—H15	119.8
O3—C2—C3	115.79 (17)	C11—C15—H15	119.8
C8—C3—C5	118.74 (18)	N1—C16—H16A	109.5
C8—C3—C2	119.06 (17)	N1—C16—H16B	109.5
C5—C3—C2	122.18 (17)	H16A—C16—H16B	109.5
C5—C4—C7	121.14 (18)	N1—C16—H16C	109.5
C5—C4—H4	119.4	H16A—C16—H16C	109.5
C7—C4—H4	119.4	H16B—C16—H16C	109.5
C4—C5—C3	119.97 (18)	N1—C17—H17A	109.5
C4—C5—C1	119.38 (17)	N1—C17—H17B	109.5
C3—C5—C1	120.24 (17)	H17A—C17—H17B	109.5
C8—C6—C7	119.81 (18)	N1—C17—H17C	109.5
C8—C6—C10	120.10 (17)	H17A—C17—H17C	109.5
C7—C6—C10	119.93 (17)	H17B—C17—H17C	109.5
			
O6^ii^—Ag1—O1—C1	31.61 (18)	C10—C6—C7—C9	−5.6 (3)
O3^i^—Ag1—O1—C1	−155.65 (17)	C7—C6—C8—C3	0.1 (3)
Ag2^i^—Ag1—O1—C1	88.4 (2)	C10—C6—C8—C3	−175.26 (19)
O5^iii^—Ag2—O4—C2	43.7 (3)	C5—C3—C8—C6	−2.1 (3)
O7^iv^—Ag2—O4—C2	177.18 (18)	C2—C3—C8—C6	176.43 (19)
Ag1^i^—Ag2—O4—C2	8.66 (17)	Ag1^ii^—O6—C9—O5	1.1 (3)
Ag1—O1—C1—O2	−150.04 (16)	Ag1^ii^—O6—C9—C7	−179.62 (13)
Ag1—O1—C1—C5	28.3 (3)	Ag2^v^—O5—C9—O6	11.0 (3)
Ag2—O4—C2—O3	−2.3 (3)	Ag2^v^—O5—C9—C7	−168.32 (13)
Ag2—O4—C2—C3	177.08 (13)	C4—C7—C9—O6	120.2 (2)
Ag1^i^—O3—C2—O4	−9.4 (3)	C6—C7—C9—O6	−57.5 (3)
Ag1^i^—O3—C2—C3	171.21 (13)	C4—C7—C9—O5	−60.4 (3)
O4—C2—C3—C8	−119.5 (2)	C6—C7—C9—O5	121.9 (2)
O3—C2—C3—C8	60.0 (3)	Ag2^iv^—O7—C10—O8	151.16 (17)
O4—C2—C3—C5	59.0 (3)	Ag2^iv^—O7—C10—C6	−27.2 (2)
O3—C2—C3—C5	−121.6 (2)	C8—C6—C10—O7	132.8 (2)
C7—C4—C5—C3	−1.1 (3)	C7—C6—C10—O7	−42.6 (3)
C7—C4—C5—C1	171.50 (18)	C8—C6—C10—O8	−45.6 (3)
C8—C3—C5—C4	2.6 (3)	C7—C6—C10—O8	139.0 (2)
C2—C3—C5—C4	−175.89 (19)	C16—N1—C11—C15	−178.3 (2)
C8—C3—C5—C1	−170.00 (18)	C17—N1—C11—C15	1.6 (3)
C2—C3—C5—C1	11.5 (3)	C16—N1—C11—C14	1.2 (4)
O1—C1—C5—C4	−138.5 (2)	C17—N1—C11—C14	−178.8 (2)
O2—C1—C5—C4	39.9 (3)	C13—N2—C12—C15	−1.2 (4)
O1—C1—C5—C3	34.1 (3)	C12—N2—C13—C14	0.9 (4)
O2—C1—C5—C3	−147.48 (19)	N2—C13—C14—C11	0.3 (4)
C5—C4—C7—C6	−0.8 (3)	N1—C11—C14—C13	179.2 (2)
C5—C4—C7—C9	−178.56 (19)	C15—C11—C14—C13	−1.2 (3)
C8—C6—C7—C4	1.3 (3)	N2—C12—C15—C11	0.2 (4)
C10—C6—C7—C4	176.73 (18)	N1—C11—C15—C12	−179.5 (2)
C8—C6—C7—C9	179.04 (19)	C14—C11—C15—C12	0.9 (3)

Symmetry codes: (i) −*x*+1, −*y*+1, −*z*+2; (ii) −*x*+1, −*y*, −*z*+1; (iii) *x*, *y*+1, *z*+1; (iv) −*x*+1, −*y*, −*z*+2; (v) *x*, *y*−1, *z*−1.

### Hydrogen-bond geometry (Å, °)

**Table:**

*D*—H···*A*	*D*—H	H···*A*	*D*···*A*	*D*—H···*A*
N2—H···O7^vi^	0.84	1.88	2.720 (2)	177
O2—H2···O8^vii^	0.82	1.73	2.541 (2)	173

Symmetry codes: (vi) *x*−1, *y*+1, *z*; (vii) *x*−1, *y*, *z*.
